# Moiré-fringeless Transparent Conductive Films with a Random Serpentine Network of Medium-Field Electrospun, Chemically Annealed Silver Microfibres

**DOI:** 10.1038/s41598-019-47779-0

**Published:** 2019-08-02

**Authors:** Dong-Youn Shin, Eun-Hye Park, Ka-Hyun Kim

**Affiliations:** 1 Department of Graphic Arts Engineering, Pukyong National University, 45, Yongso-ro, Nam-gu, Busan 48513 Republic of Korea; 2 Department of Physics, Chungbuk National University, Chungdae-ro 1, Seowon-gu, Cheongju-si, Chungcheongbuk-do 28644 Republic of Korea

**Keywords:** Electronic devices, Electrical and electronic engineering, Mechanical engineering

## Abstract

Low-cost flexible transparent conductive films (TCFs) with direct writing of metal grids have been explored as a promising alternative to conventional indium-tin-oxide-based TCFs for future flexible electronics. However, flexible TCFs have raised technical concerns because of their disadvantages, such as low resolution, low productivity, poor optoelectrical performance, poor thermal stability, and adverse moiré fringes, which primarily arise from the superposition of periodic patterns. Herein, a facile and highly productive method to fabricate moiré-fringeless TCFs with good optoelectrical characteristics and excellent thermal stability is presented using a single-pass printed random serpentine network of medium-field electrospun silver microfibres (AgMFs) with a line width of 2.32 ± 0.97 μm by exploiting the random serpentine motion of medium-field electrospinning, enabling moiré-fringeless TCFs. The electrical in-plane anisotropy of the TCFs can be kept well below 110.44 ± 1.26% with the *in situ* junction formation of the AgMFs in the transverse direction. Combined thermal and chemical annealing of the AgMFs enables high productivity by reducing the thermal annealing time by 40%. The good optoelectrical performance, fair electrical in-plane anisotropy, high productivity, and superior thermal stability of the TCFs with the single-pass printed random serpentine network of medium-field electrospun AgMFs are suitable properties for flexible electronics such as ultra-large digital signage with LEDs.

## Introduction

The industrial demand for low-cost, high-volume production of flexible transparent conductive films (TCFs) has grown, as various optoelectronic appliances like photodetectors^1–3^, memristors^4^, photovoltaic devices^5^, and organic light-emitting diodes^6^ require flexibility, high transparency, and electrical conductivity; hence, TCF alternatives to indium-tin-oxide (ITO)-based TCFs, which generally require costly photolithographic processes *in vacuo*, have been sought. One group of alternative TCFs is based on the application of a conductive material on a flexible substrate, such as conducting polymers^7^, highly metallic single-walled carbon nanotubes^8^, graphene flakes^9^, or silver nanowires (AgNWs)^10^. However, the low electrical conductivity due to either the limited carrier mobility of a conducting polymer or the high inter-tube/layer junction resistance of carbon nanotubes or graphene platelets prevents achievement of the industrial requirements for highly conductive TCFs. The poor thermal stability of AgNWs is also a hurdle preventing their industrial applications^11^.

The other group of alternative TCFs with only metallic grids or metallic grids and a subsequent coating of a conducting material has been attempted to further improve the electrical conductivity. For the direct construction of metallic grids fine enough to be invisible to the naked eyes, a variety of printing techniques have been explored, as shown in Supplementary Fig. S1. Their intrinsically additive nature, i.e., depositing valuable conductive materials only where necessary, is expected to lower the production cost of TCFs. Gravure printing with an engraved plate (Supplementary Fig. S1a)^12^, flexographic printing with a relief plate (Supplementary Fig. S1b)^13^, and inkjet printing (Supplementary Fig. S1c) have been considered for high-volume production of TCFs. However, these techniques are generally not suitable for high-volume production of TCFs with metallic grid line widths below 10 μm, albeit there might be some exceptions such as inkjet printing, which can fabricate invisible silver tracks in an interesting manner using either a capillary force in hot-embossed trenches^14^ or the coffee-ring effect^15,16^.

Reverse-offset printing shown in Supplementary Fig. S2 might be considered as another method for mass production of TCFs with metallic grids at a micrometric resolution as fine as 1 µm to 3.6 µm^17,18^. However, the superiority of reverse-offset printing is attained at the cost of a considerable waste of metallic ink in the off process, which might severely undermine the economical benefit of the printing technique. In fact, more than 90% of the metallic ink on the blanket roller needs to be removed in the off process of reverse-offset printing to acquire TCFs with an optical transmittance of 90%, resulting in the innate disadvantage of the technique. Moreover, the cleaning and contamination of the cliché and the degradation of the blanket material due to the solvent components of the ink impede the application of reverse-offset printing in industrial production lines^19^.

Electrohydrodynamic (EHD) jet printing has drawn attention due to its versatility in terms of a wide range of printable viscosities^20^, various printing modes such as drop-on-demand, continuous jet, and spraying, as shown in Supplementary Fig. S3, and its capability to directly create patterns at a micrometric or even sub-micrometric resolution^21–23^ with enhanced electro-mechanical contact performance^24^. Although the drop-on-demand mode of EHD jet printing has demonstrated great capability in constructing sub-micrometric 2D or 3D patterns^22,23^, it is unfit for mass production because its droplet generation rate is lower than that of inkjet printing, which implies low productivity. Imprecise deposition of electrically charged droplets is problematic because the electrical charge distribution of a substrate is likely to be localized by a non-uniform distribution of electrically conductive and dielectric components. The instrument also requires an extremely small nozzle, e.g., with an outer diameter of approximately 1 µm to 1.8 µm^22^, to construct sub-micrometric features, which compromises the substantial benefit of EHD jet printing in which ultra-fine features can be created using a large nozzle without clogging.

Despite the many modes of EHD jet printing, its practical applications are limited to the simple, continuous jet printing mode. However, the construction of metallic grids for TCFs in continuous EHD jet printing mode at a short stand-off distance before the onset point of the bending instability of the jet, which is hereafter referred to as near-field electrospinning, generally requires double-pass printing using either a square or an orthogonal sinusoidal pattern, as shown in Supplementary Fig. S4. The complexity of the printing system for double-pass printing prevents near-field electrospinning from achieving high-speed roll-to-roll production of flexible TCFs. Moreover, the appearance of moiré fringes due to the superposition of periodic patterns is a challenging issue in the art of fabricating TCFs.

The continuous EHD jet printing mode at a long stand-off distance far away from the onset point of the bending instability of the jet^25^, which is hereafter referred to as far-field electrospinning, is regarded as a much simpler process to produce a metallic network in a random manner with single-pass printing^26,27^. However, the random whipping motion of far-field electrospinning is uncontrollable, and the as-spun metallic network in a roll-to-roll system is prone to have non-uniform fibre spatial density^28,29^. Multiple depositions of electrospun metallic networks to assure spatial uniformity occasionally exacerbates the problem, which is a concern given the need for TCFs with high optoelectrical uniformity.

As of now, the existing production methods for TCFs do not fulfil the aforementioned requirements all at once, i.e., high electrical conductivity, good thermal stability, invisibility, high productivity, and controllability in spatial density. Herein, we present a novel alternative process for fabricating moiré-fringeless TCFs with single-pass printing for high-volume roll-to-roll production by exploiting the random serpentine motion of medium-field electrospinning at a stand-off distance near the onset point of the bending instability of the jet. It is intended to overcome the existing drawbacks of conventional production methods for TCFs. The influence of the random serpentine network of electrospun silver microfibres (AgMFs) on the optoelectrical performance and thermal stability of the TCFs is investigated and experimental results are presented on the combined thermal and chemical annealing^30,31^ of AgMFs to enable high productivity. In the end, the benefits of medium-field electrospun AgMFs for ultra-large digital signage are discussed.

## Results and Discussion

### Morphological characteristics of electrospun AgMFs

**Figure 1:**
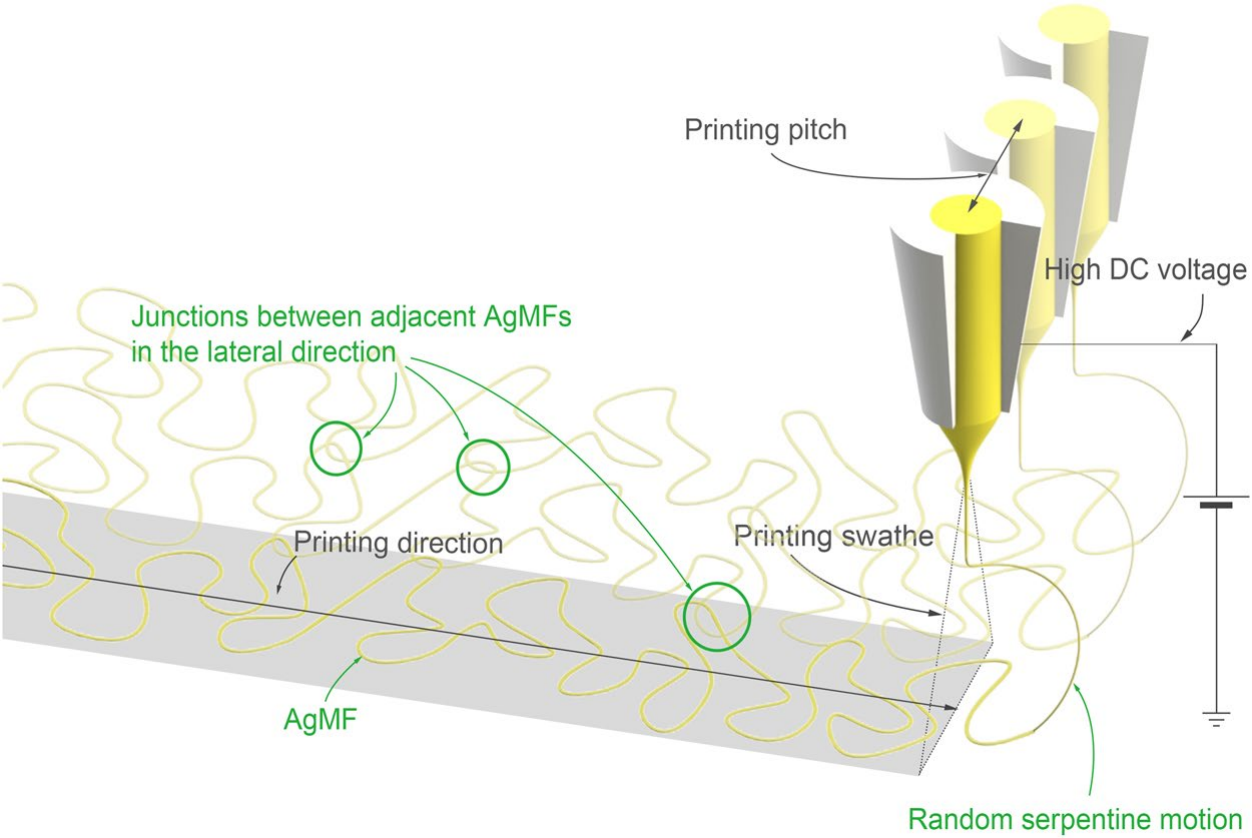
The printing scheme to construct a TCF with the single-pass printed random serpentine network of medium-field electrospun AgMFs.

**Figure 2:**
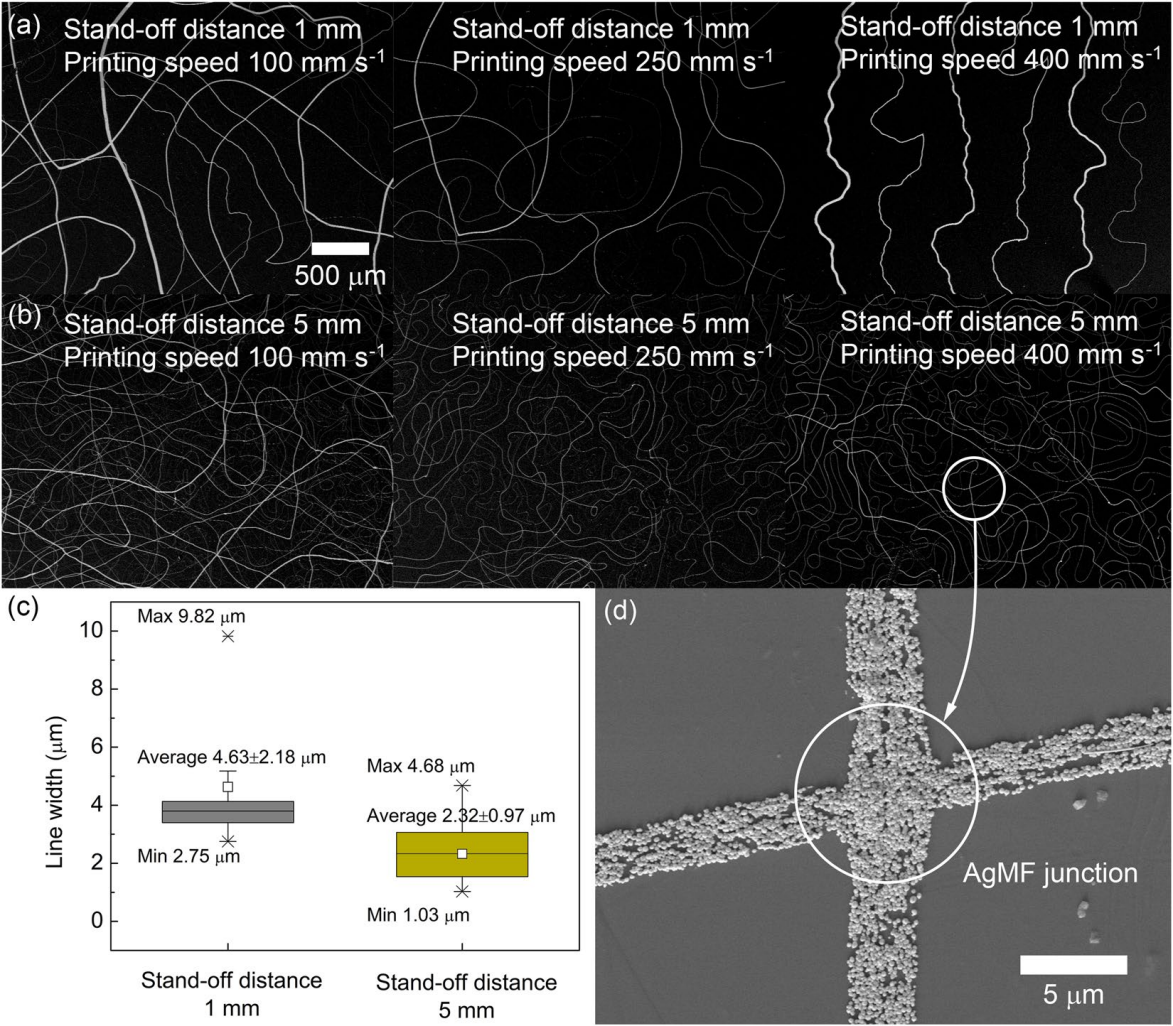
(**a**) Representative SEM micrographs of electrospun AgMFs at stand-off distances of 1 mm and (**b**) 5 mm for a printing speed range from 100 mm s^−1^ to 400 mm s^−1^, (**c**) a statistical plot of the measured line width, and (**d**) a representative SEM micrograph of well-fused AgMF junctions.

Although the randomness of the far-field electrospun AgMFs far beyond the onset point of the bending instability of the jet is anticipated to assure statistical spatial uniformity, the non-uniform electric field distribution arising from the localized electrical charge accumulation on the substrate results in a non-uniform spatial density of AgMFs^32^, which remains a challenge in the practical application of the far-field electrospinning. On the contrary, medium-field electrospinning, which lies at the modal transition between far-field and near-field electrospinning, has better control over spatial density than far-field electrospinning, since a shorter stand-off distance forces the electrospun AgMFs to land within the range of the printing swathe, as shown in Fig. 1 before they are largely deflected by the uneven electric field distribution over the substrate. Moreover, the deposited pattern of the AgMFs is rather controllable, as shown in Fig. 2a,b. In general, the electrospun AgMFs transition from a linear pattern to a serpentine pattern as the stand-off distance increases and as the printing speed decreases. The line width of the medium-field electrospun AgMFs is more affected by the stand-off distance than the printing speed. By simply increasing the stand-off distance from 1 mm to 5 mm, the average line width of the AgMFs decreases from 4.63 ± 2.18 μm to 2.32 ± 0.97 μm by 49.89%, as shown in Fig. 2c. The narrower line width at a stand-off distance of 5 mm compared to that at a stand-off distance of 1 mm is anticipated, as AgMFs electrospun at a farther stand-off distance undergo more stretching due to the accumulated electrical charge of the jet experiencing a longer flight period before reaching the substrate^32^, which also favourably reduces the standard deviation of the AgMF line width. The *in situ* junction fusion of the AgMFs during medium-field electrospinning contributes to a low junction resistance, as shown in Fig. 2d, in comparison to AgNWs.

### Optoelectrical characteristics and thermal stability of electrospun AgMFs

**Figure 3:**
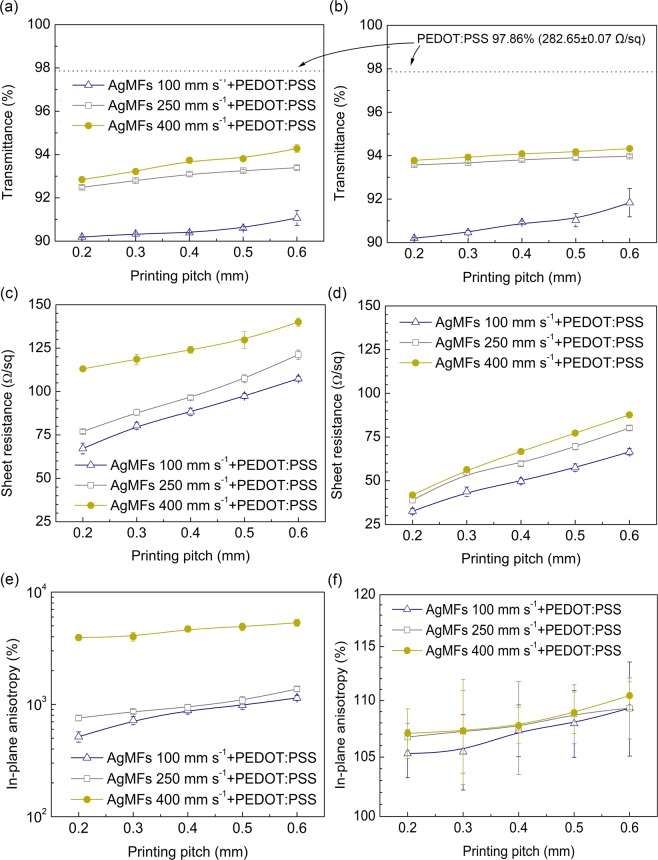
Optical transmittance of the TCFs with electrospun AgMFs at stand-off distances of (**a**) 1 mm and (**b**) 5 mm and the subsequent wire-bar-coated layer of PEDOT:PSS. Sheet resistance at stand-off distances of (**c**) 1 mm and (**d**) 5 mm and electrical in-plane anisotropy at stand-off distances of (**e**) 1 mm and (**f**) 5 mm.

The optical transmittance of the TCFs with medium-field electrospun AgMFs and the subsequent wire-bar-coated layer of PEDOT:PSS is plotted in Fig. 3. The reduction in the optical transmittance with a narrower printing pitch is comprehensible since the deposition of more AgMFs certainly impedes light transmission. In addition, the deposition of more AgMFs at the slowest printing speed of 100 mm s^−1^ apparently contributes to the low optical transmittance. The optical transmittance abruptly increases at a printing speed of 250 mm s^−1^ and exhibits no conspicuous difference at a printing speed of 400 mm s^−1^. The linear pattern of the AgMFs appearing at a higher printing speed is conjectured to occupy a smaller TCF area than the serpentine pattern of the AgMFs appearing at a low printing speed, as shown in Fig. 2a. Accordingly, TCFs with a more serpentine-like pattern of AgMFs printed at a stand-off distance of 5 mm might exhibit a lower optical transmittance than those printed at a stand-off distance of 1 mm. However, the noticeable reduction in the line width of the AgMFs at a stand-off distance of 5 mm, i.e., 49.89% decrease relative to the line width at a stand-off distance of 1 mm, as shown in Fig. 2c, prominently compensates for the increased areal occupancy of a TCF by the serpentine pattern of the AgMFs, which might lead to an imperceptible difference in the optical transmittance for stand-off distances of 1 mm and 5 mm, as shown in Fig. 3b.

The sheet resistance of TCFs with a serpentine pattern appearing at a stand-off distance of 5 mm tends to be lower than that of TCFs with a linear or serpentine pattern appearing at a stand-off distance of 1 mm, as shown in Fig. 3c,d. The serpentine pattern created at a stand-off distance of 5 mm greatly increases the population of AgMF junctions with adjacent AgMFs, as shown in Fig. 2b, consequently providing more electrical pathways. Consequently, a noticeable difference in the sheet resistance for stand-off distances of 1 mm and 5 mm appears, which differs from the case of optical transmittance. The above hypothetical elucidation is corroborated by the results of the electrical in-plane anisotropy of the TCFs, herein defined as the ratio of the electrical resistance in the transverse direction to that in the printing direction. As shown in Fig. 3e,f, the lack of AgMF junctions at a stand-off distance of 1 mm and a printing speed of 400 mm s^−1^ results in a large electrical in-plane anisotropy up to 5334.65 ± 372.83%. As the population of AgMF junctions increases with a lower printing speed of 100 mm s^−1^, the electrical in-plane anisotropy decreases to 515.22 ± 53.95%. On the other hand, the electrical in-plane anisotropy of the TCFs at a stand-off distance of 5 mm remains well below 110.44 ± 1.26% for all experimental conditions, clearly indicating the dominance of the population of AgMF junctions over the sheet resistance of the TCFs.

**Figure 4:**
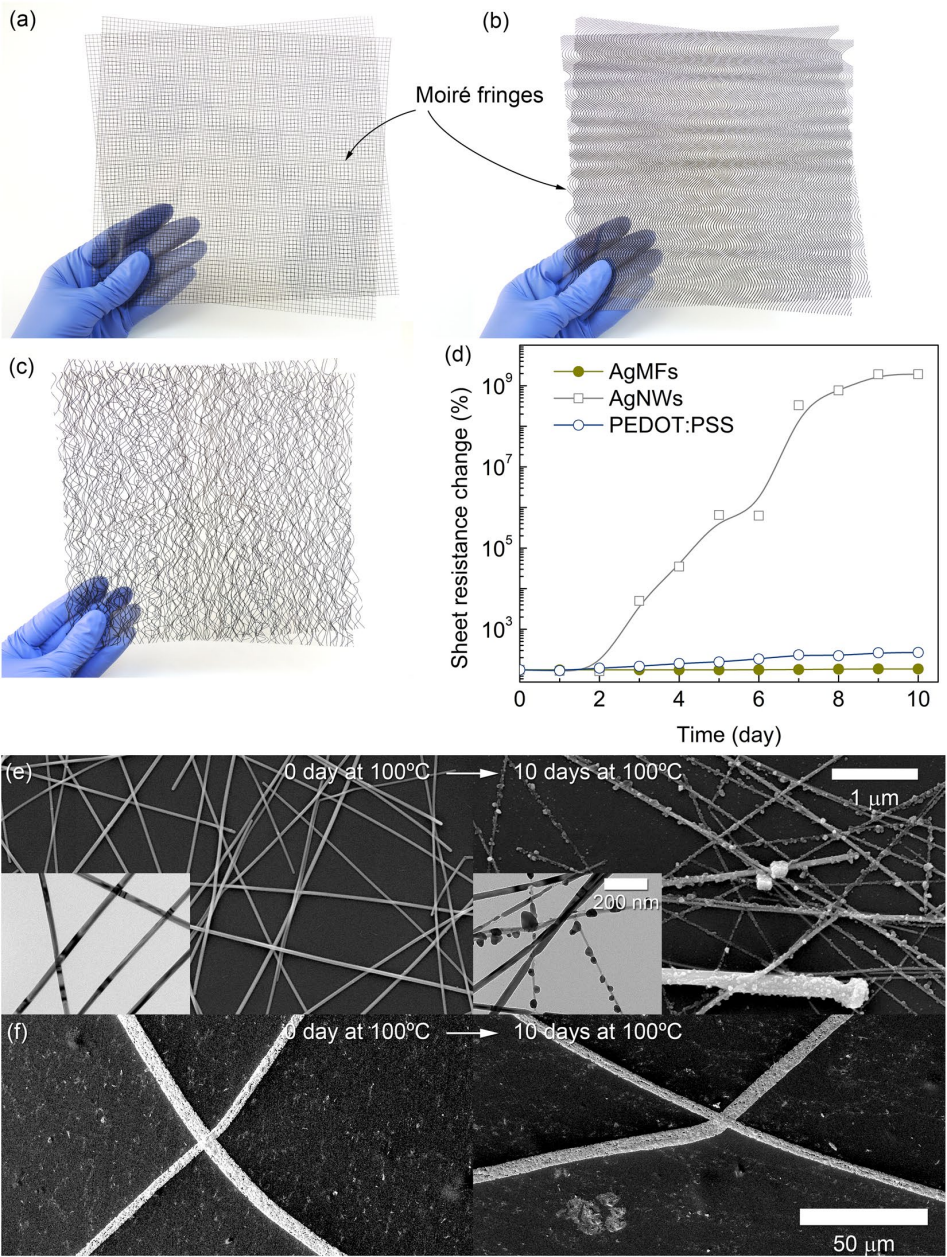
Appearance of moiré fringes due to the superposition of periodic (**a**) square and (**b**) sinusoidal patterns, and (**c**) the absence of moiré fringes due to the superposition of random serpentine patterns. Temporal changes in the sheet resistance of TCFs with AgMFs, AgNWs, and PEDOT:PSS in (**d**). Morphological changes of AgNWs and AgMFs over 10 days on a hot plate at 100 °C in (**e**,**f**), respectively.

In addition to the facile production of TCFs with better control over the spatial density than far-field electrospinning, the benefits of a random serpentine network of medium-field electrospun AgMFs include its moiré-fringeless nature and excellent thermal stability. A superposition of periodic patterns or even a superposition of identical random patterns is likely to cause moiré fringes, as shown in Fig. 4a,b (Supplementary Fig. S5 for the presence and absence of moiré fringes of TCFs with periodic and random serpentine patterns, respectively). However, the nature of the random serpentine network of medium-field electrospun AgMFs does not lead to moiré fringes in the TCF, as shown in Fig. 4c. The micrometric scale of the AgMFs also results in a thermal stability that is much larger in the AgMFs than the AgNWs, as shown in Fig. 4d, where the sheet resistance of the AgNWs increases up to 1.92 × 10^9^% over 10 days at 100 °C due to structural disintegration (Fig. 4e,f for TEM and SEM images of AgNWs and AgMFs before and after thermal stability tests), whereas the sheet resistance of the AgMFs remains well below 105 ± 3.25%.

### Combined thermal and chemical annealing of electrospun AgMFs

**Figure 5:**
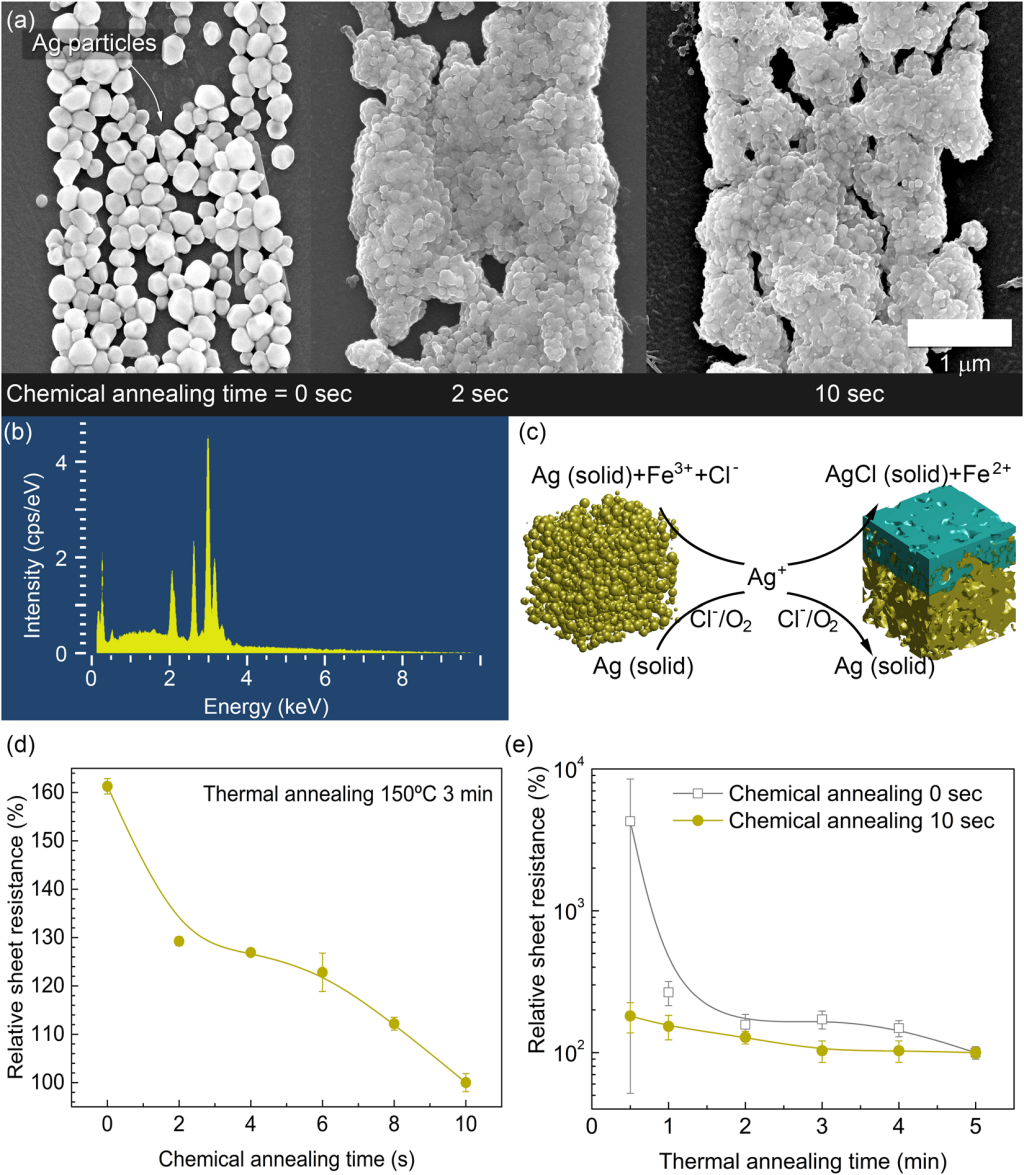
SEM micrographs and EDS spectra of chemically annealed AgMFs in (**a**–**c**) illustration of reactions during chemical annealing, (**d**) impact of the chemical annealing time on the relative sheet resistance, and (**e**) impact of combined thermal and chemical annealing on the relative sheet resistance.

**Figure 6:**
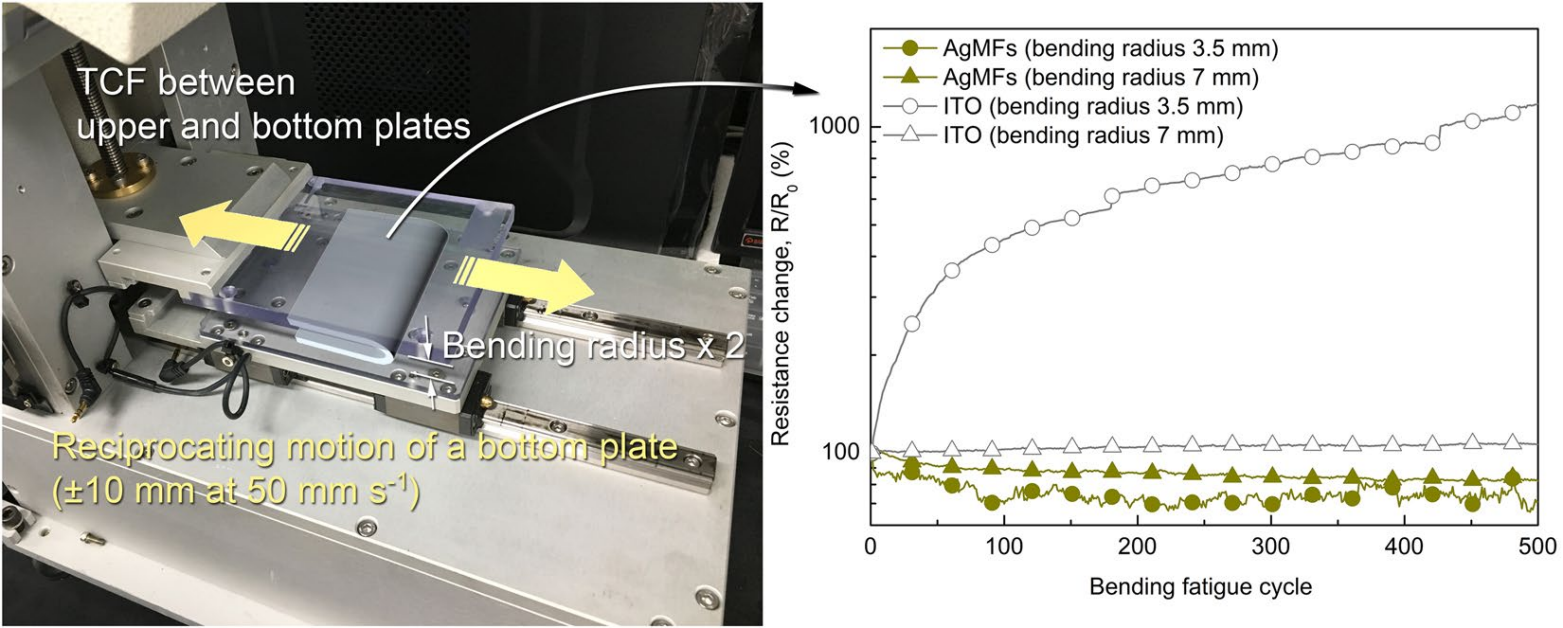
*In-situ* measured temporal changes in the electrical resistance of TCFs during inner bending fatigue testing.

In general, silver paste with silver sub-micrometric particles is likely to have such benefits as relatively cheaper material cost, easier dispersion in a liquid vehicle, lower viscosity with less interaction between particles^33^, compared to that with nanoparticles. However, the requirement of a higher sintering temperature of silver sub-micrometric particles than silver nanoparticles does not fit for applications like flexible TCFs, which utilize polymeric substrates with low glass transition temperatures^34^_._ Silver sub-micrometric particles of as-electrospun AgMFs were also found to be discretely laid down rather than well fused each other even after sintering, as shown in Fig. 5a. The minimum possible line width of AgMFs with silver sub-micrometric particles was consequently bound to be a few micrometres, below which AgMFs were likely to be discontinuous (Supplementary Fig. S6) and resulted in TCFs with poor optoelectrical performances. To expedite the sintering and interconnection of discrete silver sub-micrometric particles, combined thermal and chemical annealing was applied by dipping the specimens in an aqueous ferric chloride solution after thermal annealing. The self-catalytic addition of silver ions on the surface of unsintered AgMFs facilitates the diffusion of silver sub-micrometric particles^30,31^, as shown in Fig. 5a. However, a non-conductive silver chloride layer was also formed on the surface of AgMFs in the course of chemical annealing, as can be seen in Fig. 5b,c, and hence too much exposure to chemical annealing would increase the sheet resistance of TCFs. Through the tedious experiments shown in Fig. 5d,e, no significant improvement in the sheet resistance was found by thermal annealing alone for longer than 5 min at 150 °C or by chemical annealing for longer than 10 sec after thermal annealing for 3 min at 150 °C. *In-situ* measured temporal changes in the electrical resistance of conventional ITO-based and AgMF-based TCFs are plotted in Fig. 6, where TCFs with AgMFs exhibit a superior bending fatigue performance to ITO-based ones.

**Figure 7:**
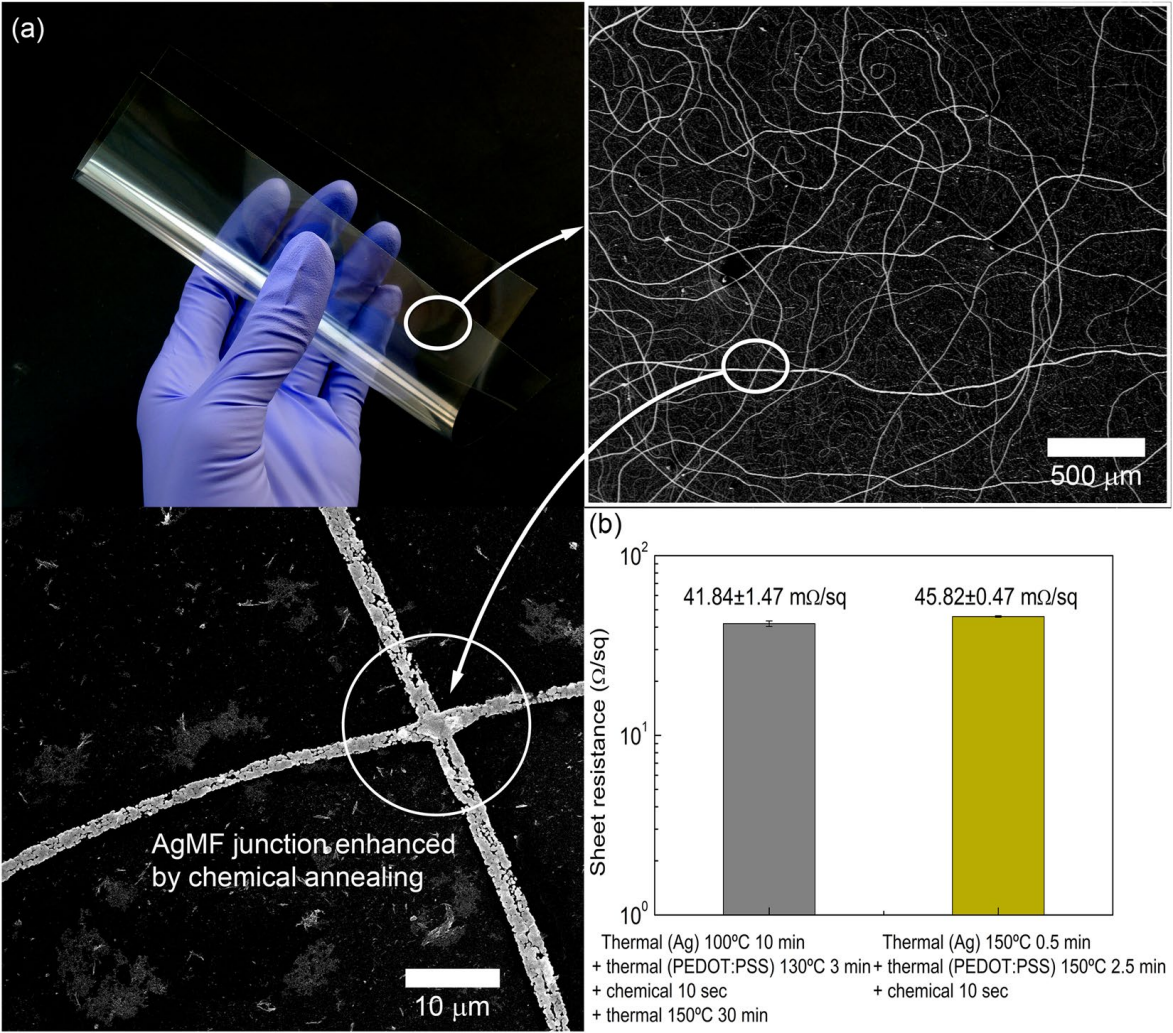
(**a**) TCF with a random serpentine network of medium-field electrospun AgMFs and (**b**) comparison of the sheet resistance without and with optimal thermal and chemical annealing steps.

To demonstrate the practical aspects of a random serpentine network of medium-field electrospun AgMFs and combined thermal and chemical annealing, TCFs as large as 15 cm × 15 cm, as shown in Fig. 7a, were fabricated with a stand-off distance, printing speed, and printing pitch of 5 mm, 400 mm s^−1^, and 0.2 mm, respectively. The measured sheet resistance of the TCFs was 45.82 ± 0.47 Ω/sq, slightly higher than the value of 41.84 ± 1.47 Ω/sq measured for the samples with a much longer thermal annealing time, as compared in Fig. 7b. The current optoelectrical properties of TCFs with a random serpentine network of AgMFs are compared with those from literatures in Supplementary Fig. S7 ^35^. TCFs have been utilized in a wide variety of electronic devices, such as displays, touch screens/sensors, photovoltaic devices, and thermal heaters/defrosters.

**Figure 8:**
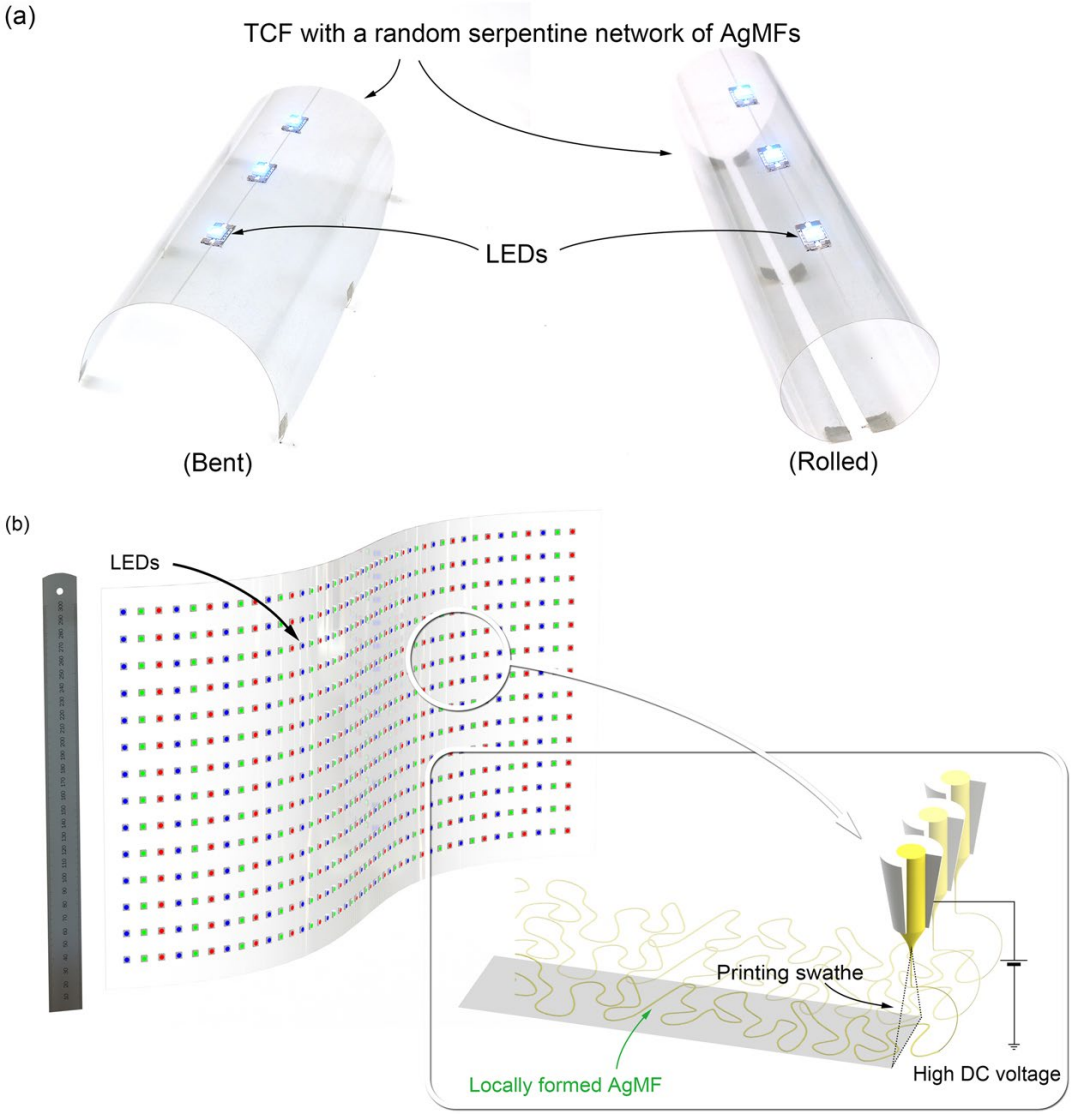
(**a**) A proof-of-concept transparent and rollable LED display for ultra-large digital signage and (**b**) conceptual illustration of ultra-large digital signage with localized transparent electrodes using medium-field electrospun AgMFs.

We attempted to extend the application of TCFs to a transparent and rollable LED display for ultra-large digital signage, and a proof-of-concept example was developed, as shown in Fig. 8a. Conventional TCFs are isotropically conductive in general since they are usually fabricated using a coating method like sputtering or wet coating with no ability to locally form an electrically conductive layer of ITO or AgNWs. The significant amount of a precious material would be wasted as the size of a TCF for digital signage becomes larger since even areas, which do not require electrical conductivity, are coated with ITO or AgNWs. Therefore, ultra-large digital signage would require to locally pattern highly conductive and transparent electrodes. As illustrated in Fig. 8b, the capability of medium-field electrospun AgMFs to selectively form transparent electrodes and locally control electrical properties by adjusting the stand-off distance might be advantageous for improving the electrical conductivity for applications in in ultra-large digital signage.

## Conclusion

In summary, a facile and highly productive method was presented to fabricate TCFs with a random serpentine network of medium-field electrospun AgMFs. A random metallic network of AgMFs with an average line width of 2.32 ± 0.97 μm was constructed with only single-pass printing, implying a higher productivity than near-field electrospinning, which generally requires double-pass printing. The optical transmittance of the TCFs was less sensitive to the stand-off distance and asymptotically approached its upper bound at a printing speed of 250 mm s^−1^ and above. In contrast, the electrical performance of the TCFs was greatly influenced by the population of AgMF junctions formed with adjacent AgMFs in the transverse direction and could be adjusted primarily by the stand-off distance and secondarily by the printing speed. The resulting optical transmittance and sheet resistance of the TCFs at the fastest printing speed of 400 mm s^−1^ and a stand-off distance of 5 mm were 93.78 ± 0.05% and 41.84 ± 1.47 Ω/sq, respectively, with an electrical in-plane anisotropy well below 110.44 ± 1.26%. The random serpentine network of medium-field electrospun AgMFs not only liberated the TCFs from moiré fringes but also resulted in a much larger thermal stability, 105 ± 3.25%, compared to that of AgNWs, 1.92 × 10^9%^, over 10 days at 100 °C. By dipping TCFs in an aqueous ferric chloride solution, the overall thermal annealing time at 150 °C decreased by 40% from 5 min to 3 min. Combined thermal and chemical annealing can realize highly productive roll-to-roll production with a shorter thermal furnace length. In the end, a proof-of-concept transparent and rollable LED display using a TCF as large as 15 cm × 15 cm was fabricated to demonstrate the practical aspects of a random serpentine network of medium-field electrospun AgMFs and combined thermal and chemical annealing. The presented method offers new opportunities for invisible electronic devices.

## Methods

### Preparation of materials

To formulate a silver paste for medium-field electrospinning, a polyethylene oxide (PEO) solution was first prepared by dissolving 2 wt% of PEO (CAS No. 25322-68-3, average M_v_ of 400000, Sigma-Aldrich Corp.) in a mixture of ethyl alcohol (CAS No. 64-17-5, Honeywell International Inc.) and deionized water (KCU-UW3, FISCO Co., Ltd.) at a weight ratio of 1 to 1 on a hot plate stirrer (SMHS-6, Daihan Scientific Co., Ltd.) with a stirring speed of 500 rpm and a temperature of 60 °C for 10 min. The prepared PEO solution was then mixed with the silver paste (solid content: 88.7 wt%, ES Ag Nano Ink, NPI Ltd.) at a weight ratio of 1 (PEO solution) to 1 (silver paste) using a planetary centrifugal mixer (ARE-310, Thinky Corp.) at a rotational speed of 2000 rpm for 10 min. The formulated silver paste for medium-field electrospinning contained silver sub-micrometric particles with an average diameter of 186.54 nm, as shown in Supplementary Fig. S8a,b.

A conducting polymer dispersion was prepared with 92.95 wt% of an aqueous dispersion of poly(3,4-ethylenedioxythiophene)-poly(styrenesulfonate) (PEDOT: PSS) (Clevios^TM^ PH 1000, Heraeus Deutschland GmbH & Co. KG), 7 wt% of ethylene glycol (CAS No. 107-21-1, Sigma-Aldrich Corp.) for an electrical conductivity enhancement^36^, and 0.05 wt% of a fluorosurfactant (FC-4430, 3 M Co.) for improved wettability on the substrate. The compounds were mixed with a hot plate stirrer at a stirring speed of 300 rpm for 10 min at room temperature.

### Preparation of samples

A random serpentine network of AgMFs was produced on a polyethylene terephthalate substrate (Dongseo Chemical Co., Ltd.) using a desktop dispensing robot (EzROBO-5 GXST2520, Iwashita Engineering, Inc.), as shown in Supplementary Fig. S9, where the formulated silver paste was delivered to a needle with inner and outer diameters of 260 μm and 460 μm, respectively, (BMN-26G, Banseok Precision Co., Ltd.) by a syringe pump (TJ-3A/W0109-1B, Longer precision pump Co., Ltd.). The printing speed of the desktop dispensing robot was set in the range of 100 mm s^−1^ and 400 mm s^−1^ with a syringe pump flow rate of 1 µL h^−1^. To explore the impact of the printing pitch on the optoelectrical performance of the experimented TCFs, the printing pitch was varied in the range of 0.2 mm and 0.6 mm. A high DC voltage was applied between the needle and the plate of the desktop dispensing robot using a high voltage generator (BT-GP-AC-30P30, Ultravolt Inc.). At stand-off distances of 1 mm and 5 mm, the applied voltages were set at 1.6 kV and 1.8 kV, respectively, which were the minimum possible voltages to achieve stable electrospinning of the AgMFs at each stand-off distance. After the specimens with electrospun AgMFs were dried on a hot plate at 100 °C for 10 min, they were coated with the PEDOT:PSS dispersion using a wire bar coater (CT-AF300VH, Coretech Korea Co., Ltd.) at a speed of 100 mm s^−1^ and then dried on a hot plate at 130 °C for 3 min. In the end, the specimens were thermally annealed on a hot plate at 150 °C for 30 min. To demonstrate an increase in productivity by combining thermal and chemical annealing, the specimens in the experimental group were dipped in an aqueous solution containing 6 wt% of ferric chloride (FeCl3, Kanto Chemical Co. Inc., Japan) up to 10 sec after thermal annealing at 150 °C up to 5 min and then compared with those in the control group described above.

For the purpose of a thermal stability comparison, specimens were prepared by spin-coating (Spin-1200D, Midas System Co., Ltd.) the prepared PEDOT:PSS dispersion and an aqueous dispersion of AgNWs (solid content: 0.3 wt%, Nanopyxis Co., Ltd.) at 2000 rpm for 2 min on microscope cover glasses (22 mm × 22 mm, DU.2355032, Duran Group Corp.), with subsequent thermal annealing on a hot plate (DHSL. HP2020300, DHSL Korea Co., Ltd.) at 150 °C for 3 min. For accelerated thermal stability testing, specimens of AgMFs, AgNWs, and PEDOT:PSS were placed on a hot plate at 100 °C for up to 10 days in an ambient environment.

## Supplementary information

Supplementary Information
