# Supplementary material for: Moiré-fringeless Transparent Conductive Films with a Random Serpentine Network of Medium-Field Electrospun, Chemically Annealed Silver Microfibres
Source: Sci Rep. 2019 Aug 2;9:11226. doi: 10.1038/s41598-019-47779-0 (PMC6677803; doi:10.1038/s41598-019-47779-0)
Supplement: Supplementary file 1 — Supplementary Information [file 41598_2019_47779_MOESM1_ESM.pdf]

Moiré-fringeless Transparent Conductive Films with a Random Serpentine Network of Medium-Field Electrospun, Chemically Annealed Silver Microfibres

Dong-Youn Shin[1,*], Eun-Hye Park[1] & Ka-Hyun Kim[2,*]

[1]Department of Graphic Arts Engineering, Pukyong National University, 45, Yongso-ro, Nam-gu, Busan, 48513, Republic of Korea. [2]Department of Physics, Chungbuk National University, Chungdae-ro 1, Seowon-gu, Cheongju-si, Chungcheongbuk-do, 28644, Republic of Korea. Correspondence and requests for materials should be addressed to D.-Y. S. (email: dongyoun.shin@gmail.com, Tel: +82-51-629-6394) or K.-Y. Kim. (email: rfpecvd@gmail.com)

Supplementary information

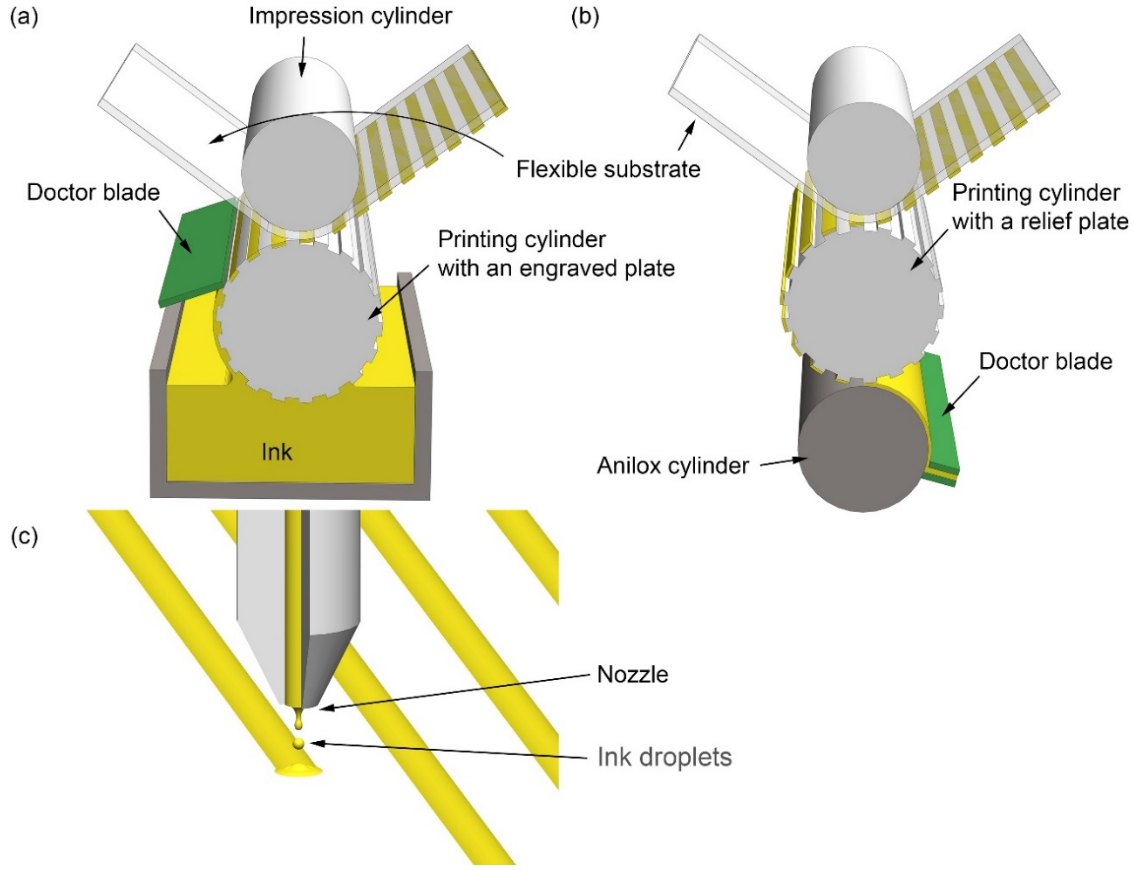

Supplementary Figure S1. Schematic illustrations of (a) gravure, (b) flexography, and (c) inkjet printing techniques.

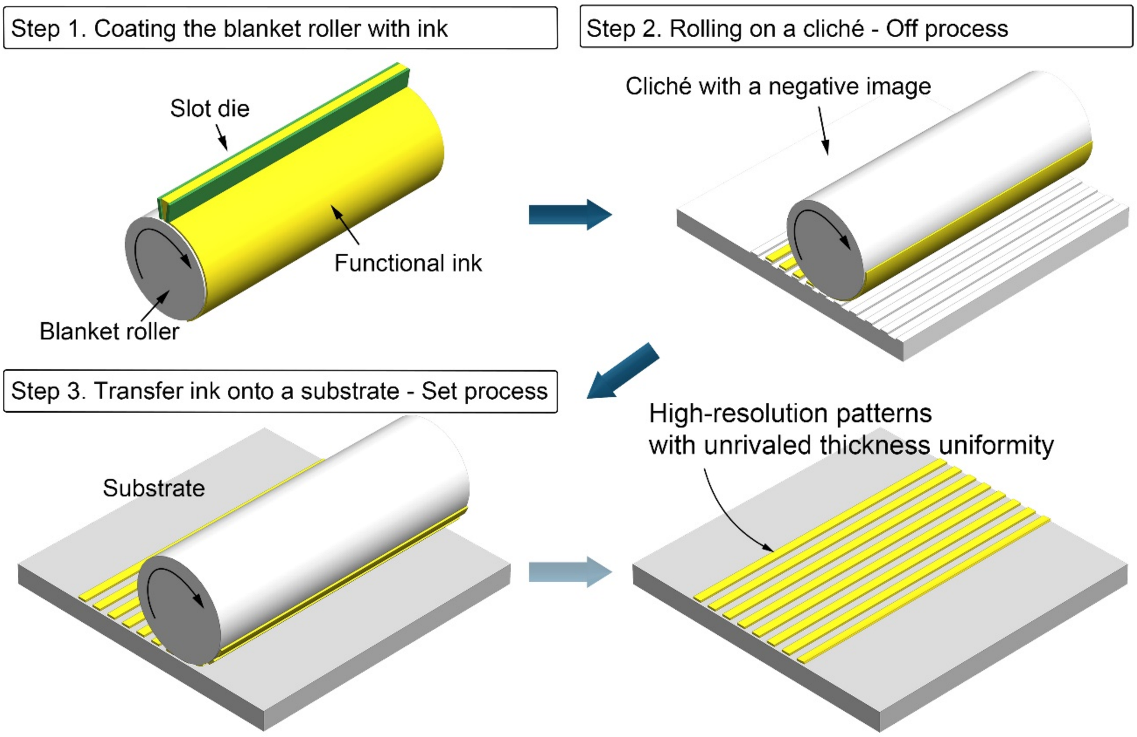

Supplementary Figure S2. Schematic illustration of a reverse-offset printing technique.

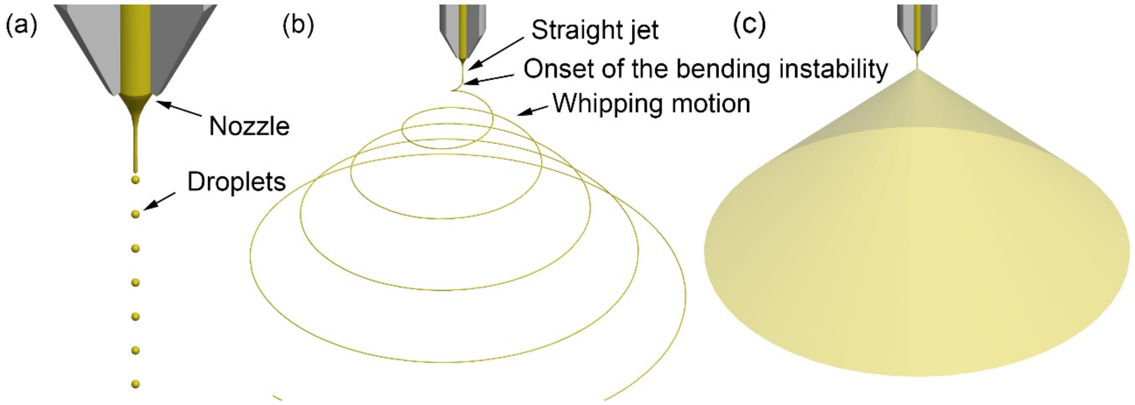

Supplementary Figure S3. Schematic illustrations of EHD jet printing modes: (a) drop-on-demand, (b) continuous jet, including spinning, and (c) spraying with minuscule droplets nebulized by electrical repulsion.

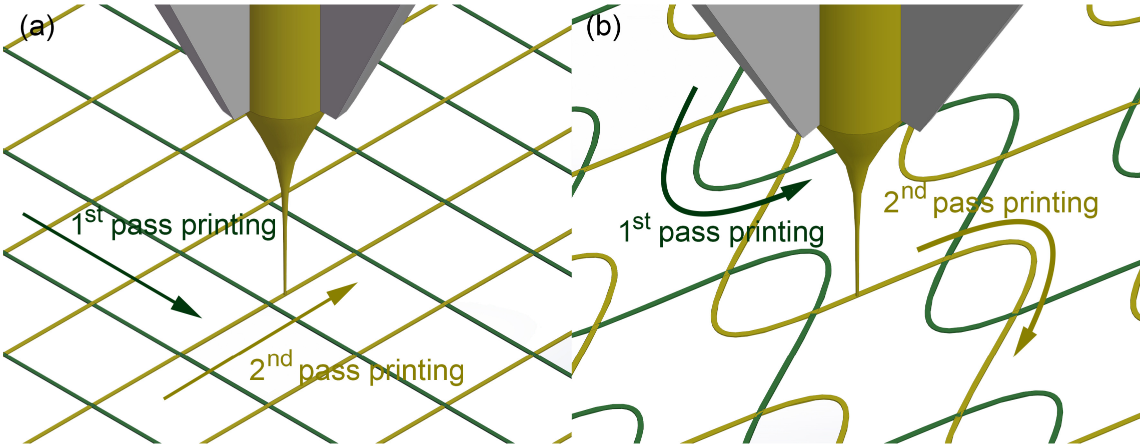

Supplementary Figure S4. Schematic illustrations of double-pass printed metallic grids using either (a) a square or (b) an orthogonal sinusoidal pattern.

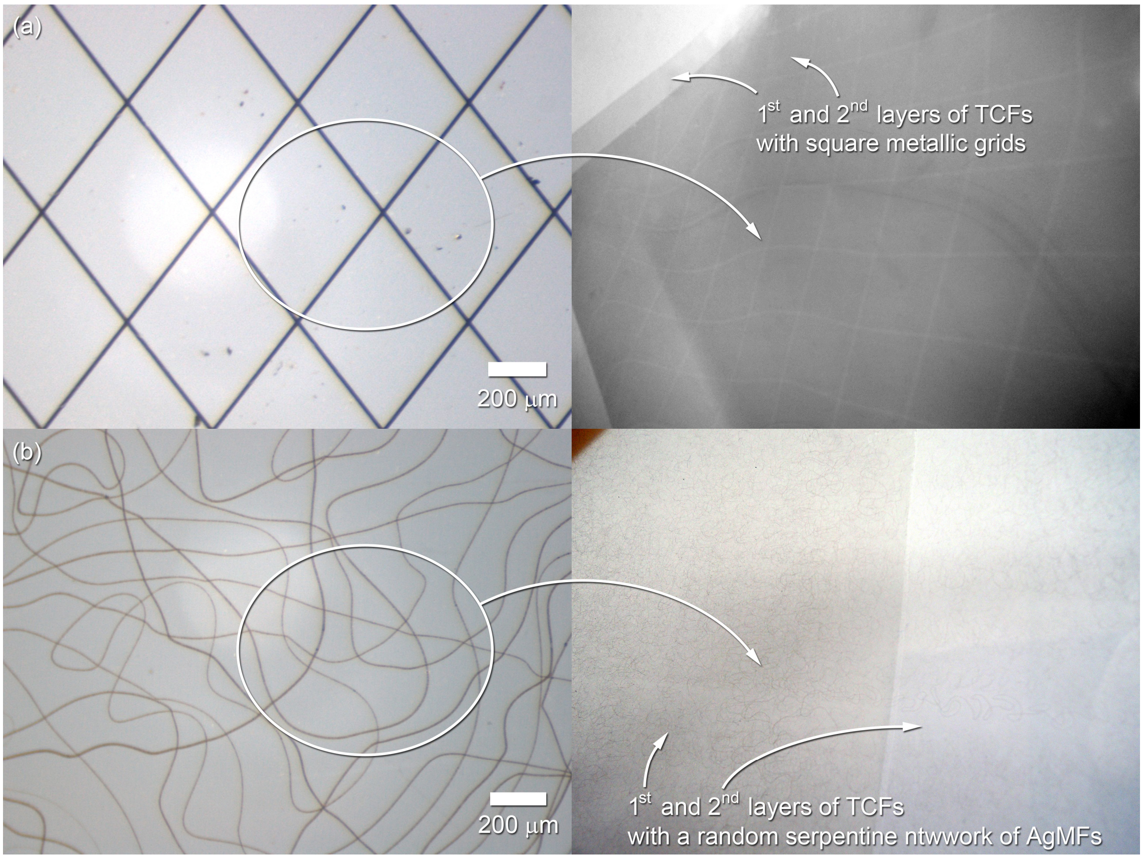

Supplementary Figure S5. (a) Appearance of moiré fringes by the superposition of conventional TCFs with periodic patterns and (b) the absence of moiré fringes even after the superposition of TCFs with random serpentine patters.

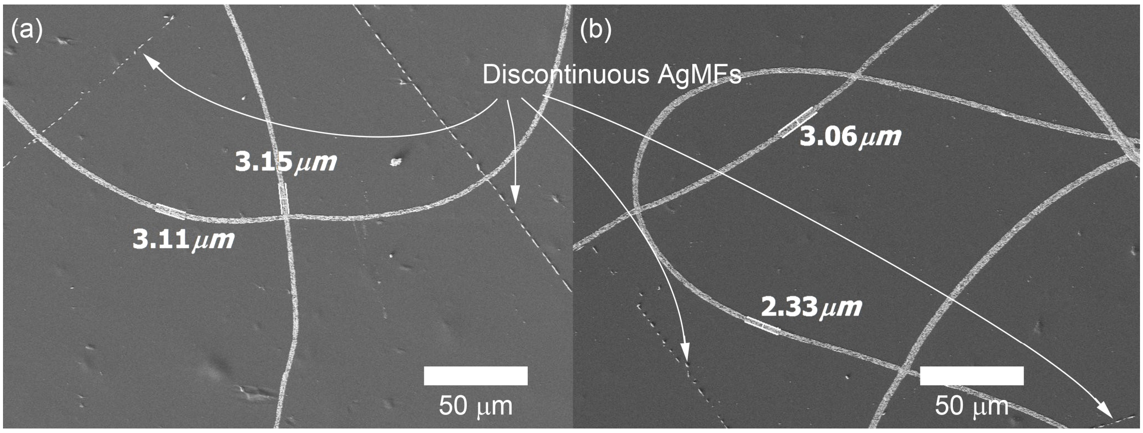

Supplementary Figure S6. SEM images of discontinuous AgMFs below one micrometre in line width at stand-off distances of (a) 1 mm and (b) 5 mm.

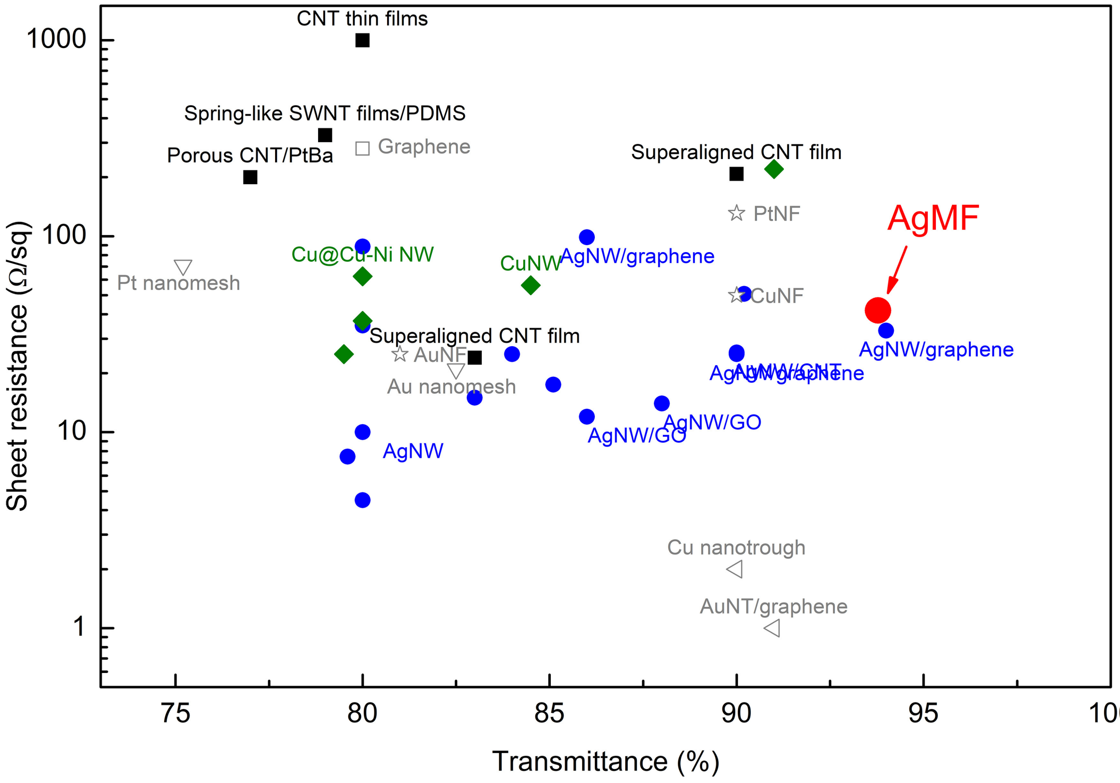

Supplementary Figure S7. Optoelectrical properties of TCFs in literatures (See Ref. 35).

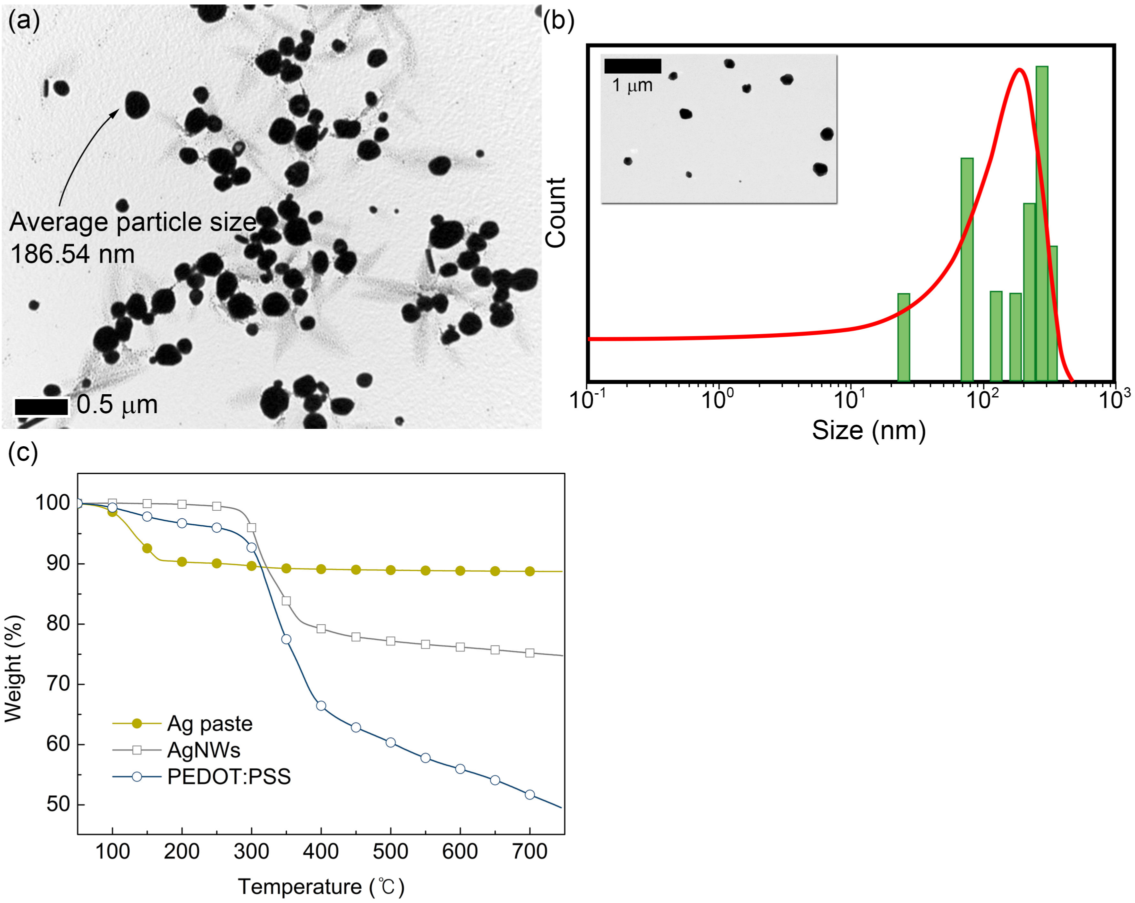

Supplementary Figure S8. (a) TEM micrograph and (b) particle size distribution of silver sub-micrometric particles in the silver paste used in the experiment, and (c) TGA thermograms of the as-received silver paste, dried forms of the silver nanowires and PEDOT:PSS.

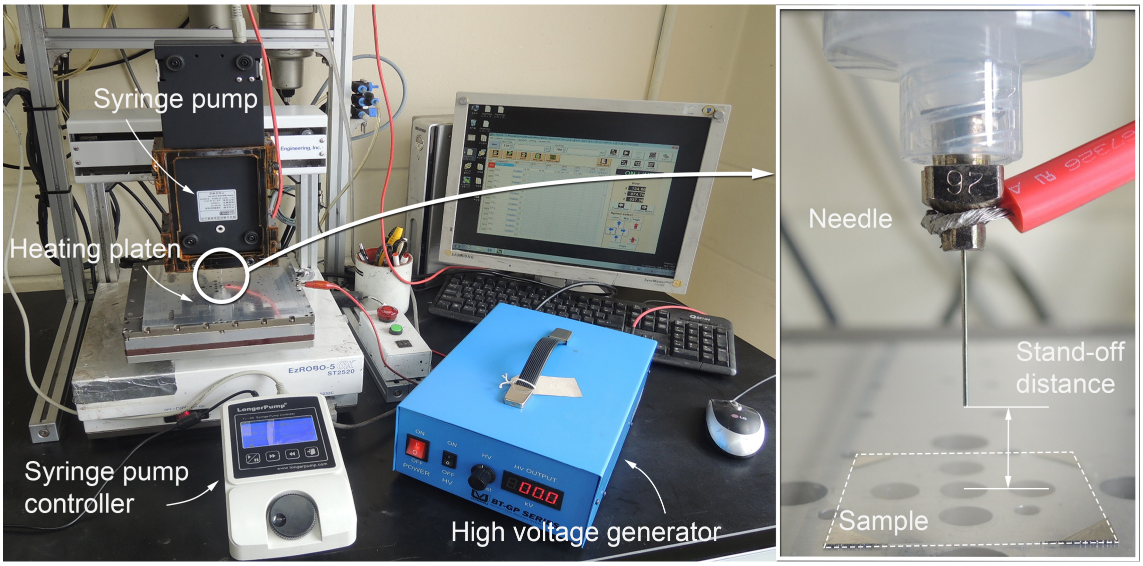

Supplementary Figure S9. Experimental setup for medium-field electrospinning.
